# Performance Analysis of the Microsoft Kinect Sensor for 2D Simultaneous Localization and Mapping (SLAM) Techniques

**DOI:** 10.3390/s141223365

**Published:** 2014-12-05

**Authors:** Kamarulzaman Kamarudin, Syed Muhammad Mamduh, Ali Yeon Md Shakaff, Ammar Zakaria

**Affiliations:** 1 Center of Excellence for Advanced Sensor Technology (CEASTech), Universiti Malaysia Perlis (UniMAP), Taman Muhibbah, Jejawi, 02600 Arau, Perlis, Malaysia; E-Mails: smmamduh@ieee.org (S.M.M.); aliyeon@unimap.edu.my (A.Y.M.S.); ammarzakaria@unimap.edu.my (A.Z.); 2 School of Mechatronics Engineering, Universiti Malaysia Perlis (UniMAP), Pauh Putra Campus, 02600 Arau, Perlis, Malaysia; 3 School of Microelectronic Engineering, Universiti Malaysia Perlis (UniMAP), Pauh Putra Campus, 02600 Arau, Perlis, Malaysia

**Keywords:** Microsoft Kinect sensor, 2D SLAM, robotics, integrated system, sensor, virtual machine, Robot Operating System

## Abstract

This paper presents a performance analysis of two open-source, laser scanner-based Simultaneous Localization and Mapping (SLAM) techniques (*i.e.*, Gmapping and Hector SLAM) using a Microsoft Kinect to replace the laser sensor. Furthermore, the paper proposes a new system integration approach whereby a Linux virtual machine is used to run the open source SLAM algorithms. The experiments were conducted in two different environments; a small room with no features and a typical office corridor with desks and chairs. Using the data logged from real-time experiments, each SLAM technique was simulated and tested with different parameter settings. The results show that the system is able to achieve real time SLAM operation. The system implementation offers a simple and reliable way to compare the performance of Windows-based SLAM algorithm with the algorithms typically implemented in a Robot Operating System (ROS). The results also indicate that certain modifications to the default laser scanner-based parameters are able to improve the map accuracy. However, the limited field of view and range of Kinect's depth sensor often causes the map to be inaccurate, especially in featureless areas, therefore the Kinect sensor is not a direct replacement for a laser scanner, but rather offers a feasible alternative for 2D SLAM tasks.

## 1. Introduction

The Simultaneous Localization and Mapping (SLAM) technique is a subject of interest in mobile robotics studies. The main idea of this technique is to leave the robot at an unknown location and let it move and build a consistent map of its surroundings. Although the problem seems straightforward, in reality it is very complex due to the non-ideal behavior of sensors and actuators. The fact that both the localization and mapping task needs input from each other to complete computation further complicates the problem [1]. The solution to the SLAM problem allows deployment of robots in many applications such as search and rescue operations, underwater surveillance and gas distribution mapping.

Numerous SLAM techniques have been developed by previous researchers utilizing different devices, including sonar sensors [2,3], cameras [4–6] and laser scanners [7–11]. However, the methods are often device-specific due to the different sensing modalities and capabilities of each device. The introduction of the Microsoft Kinect in 2010 has allowed an extension of the available SLAM methods. Most researchers take advantage of the RGB camera and the 3D depth sensor available on the Kinect to perform 3D or RGB-Depth (RGB-D) mapping [12–17]. Although the results seem promising, it requires an extensive amount of memory and processing power. Thus, 2D SLAM system may be preferred in certain applications, while still leveraging the Kinect's 3D depth sensor to detect obstacles of variable shapes and sizes.

Several researchers have attempted to perform 2D mapping using the Kinect with laser scanner-based SLAM algorithms [18–20]. However, to the authors' knowledge, the results shown are often simplified and did not consider different scenarios such as mapping in a featureless area or in the conditions where objects of variable shapes and sizes exist. Also, the effect of modifying the SLAM parameters in different environments has also not been studied previously. Such an analysis enables a more thorough evaluation of the suitability of the Kinect as a replacement sensor for 2D laser scanners.

Hence, in this paper, we analyzed the performance of two laser scanner-based SLAM algorithms (*i.e.*, Gmapping and Hector SLAM) using a Microsoft Kinect as the only navigation sensor. The techniques were selected since they are open-source, widely used and more importantly, they are based on two different approaches: Gmapping is based on Rao-Blackwellized Particle Filter (RBPF) and uses odometry for pose estimation, while the Hector SLAM is based on a Gauss-Newton approach and relies on scan matching alone to estimate the pose. The method proposed in [21] was used to convert the 3D depth data from Kinect into 2D laser scan-like obstacle locations. The performance of the system in real-time was tested in two different environments: a small room with no features and a typical office corridor with desks and chairs. Then, using the data logged from the experiments, the SLAM process was simulated repeatedly with different parameter settings, while observing any effect on the performance and accuracy of the final map.

Another contribution of this paper is the design of a system that utilized a Linux virtual machine in Windows to execute the real-time 2D SLAM algorithms. The base station of the system was installed with Windows 7 as the main operating system and Ubuntu in a virtual machine. A LabVIEW program (in Windows) was developed to control the whole system execution and provide a user interface while the Robot Operating System (ROS) was installed in the Linux virtual machine for computation of SLAM using the open-source algorithms (*i.e.*, Gmapping and Hector SLAM). The two-way communication between these two programs is achieved via the TCP/IP protocol and ROSBridge tool.

## 2. Kinect *vs.* Laser Scanners

Kinect was launched by Microsoft on November 2010. It was initially designed as a vision-based controller for the Microsoft Xbox 360 video game console. The device consists of several sensors including a RGB sensor, a 3D depth sensor, multi-array microphones and an accelerometer [22,23]. The Kinect's depth sensor (the sensor used in this research) produces depth images at 640 × 480 pixels (VGA size) [22]. The sensor has a field of view of 57° horizontally and 43° vertically. The optimal operating range of the sensor is said to be between 0.8 to 3.5 m, although several researchers claim that it can be extended up to 0.4 to 6 m. The Kinect's depth sensor is able to provide depth resolution of 1 cm and spatial x/y resolution of 3 mm at 2 m distance from the sensor. The maximal stable transfer rate of the frame is up to 30 Hz, depending on the driver or software used [22,23].

Table 1 compares the specifications of Kinect's depth sensor with respect to the general specifications of 2D laser scanners. The comparison is provided since the SLAM algorithms used in this paper (*i.e.*, Gmapping and Hector SLAM) are based on laser scanners and the sensor had been reported to work well for most robotics applications, particularly SLAM.

Referring to Table 1, the laser scanner is superior to the Kinect in terms of range as it can detect obstacles up to 250 m (certain models). In addition, the laser scanner has significantly wider horizontal angle (*i.e.*, field of view) of up to 360 degrees. However, the Kinect has advantages in terms of the extra dimensionality it provides (*i.e.*, three dimensions) and its significantly lower cost. Although the laser scanner is generally better than the Kinect in terms of range and angle, the Kinect could be more effective as a navigation sensor since it is able to provide 3D views in a relatively fast sampling period. This aspect is very important in order to perform SLAM in a real environment where there exist objects of variable shape and size. The robot will also able to avoid certain obstacles that are typically unseen by 2D sensors. Further explanations can be found in Section 3.2 and [21].

## 3. The Methods and Algorithms

### 3.1. Odometry

In this project, we were only interested in 2D localization (*i.e.*, *x*, *y* and *θ*) since the robot is employed on flat surface. The odometry technique is used to estimate the robot's pose with respect to the starting pose. However, this method is sensitive to errors since it estimates the position by integrating the velocity measurements over time. Rapid and accurate data collection, equipment calibration and efficient data processing must be done for odometry to be used effectively.

The robot used in this project uses a differential drive mechanism. It consists of two DC motors that are connected to a gear and two wheels on each side. Each motor can be independently rotated either clockwise or anticlockwise. By varying the speed and direction of the motors, the robot can move forward, backwards, rotate or move along a curve. An encoder is mounted on each motor to estimate the rotation velocity. Using this value, the velocity of the left, *V_l_* and right, *V_r_* wheel along the ground can be estimated as:

(1) $$V_{l}=\alpha_{l}r/G$$

(2) $$V_{r}=\alpha_{r}r/G$$

where *α_l_* and *α_r_* are rotational velocity of the right and left motor respectively, r is the radius of wheel and G is the gear ratio. There are three different styles of movement that the robot can exhibit using the differential mechanism; linear motion (Equation (3)) rotation about its position (Equation (4)) and moving along a curve (Equations (5) and (6)). Note that the calculation of new robot pose in each case below is relative to the starting pose after the robot moves for a period of *δt* in each time step:

(3) $$\left[x^{\prime },y^{\prime },\theta^{\prime }]=[x+\text{Vcos}\left(\theta \right)\delta t,y+\text{Vsin}\left(\theta \right)\delta t,\theta \right]$$

(4) $$\left[x^{\prime },y^{\prime },\theta^{\prime }]=[x,y,\theta +2V\delta t/l\right]$$

[*x*′, *y*′, *θ*′] and [*x*, *y*, *θ*] represent previous and current robot pose respectively, *V* takes the value of the robot's right wheel (*V_r_*) and *l* indicates the separation between the left and right wheels. In a special case where the robot moves along a curve, the center of the curvature (denoted as [*ICC_x_, ICC_y_*]) needs to be calculated first as in Equation (5):

(5) $$\left[ICC_{x},ICC_{y}\right]=\left[x-\text{Rsin}\left(\theta \right),y+\text{Rcos}(\theta )\right]$$

Then, the subsequent pose can be determined using:

(6) $$\left[\begin{array}{c} x^{\prime }\\ y^{\prime }\\ \theta^{\prime } \end{array}\right]=\left[\begin{array}{ccc} \text{cos}(\omega \delta t) & -\text{sin}(\omega \delta t) & 0\\ \text{sin}(\omega \delta t) & \text{cos}(\omega \delta t) & 0\\ 0 & 0 & 1 \end{array}\right]\left[\begin{array}{c} x-ICC_{x}\\ y-ICC_{y}\\ \theta \end{array}\right]+\left[\begin{array}{c} ICC_{x}\\ ICC_{y}\\ \omega \delta t \end{array}\right]$$

where R denotes the radius of curvature and *ω* represent the angular velocity about the *ICC*. The controller on the robot has been programmed to perform the odometry calculation before the information is transmitted to the base station for SLAM computation (*i.e.*, Gmapping).

### 3.2. Kinect's Depth Data to 2D Scan

**Figure 1.**
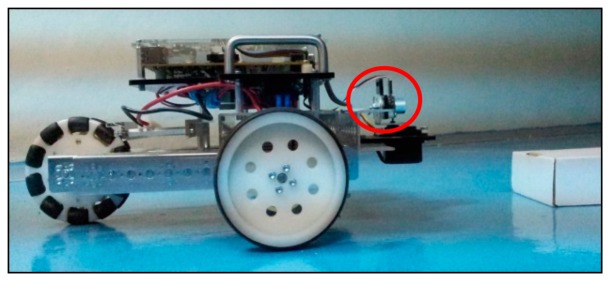
An example showing a sonar sensor placed at certain height (indicated by red circle) unable to detect and measure the obstacle location.

The use of 1D or 2D sensors such as sonar and 2D laser scanners for navigation and mapping is believed to not be efficient due to the possibility of missing or misinterpreting an obstacle's location. Figure 1 shows a scenario representing these problems. The sensor fixed at a certain height on the robot is unable to see the obstacle that is located below the visible range. Another possibility is that the sensor may mistakenly interpret an object's location if it is non-uniform in shape (e.g., L shape).

A 3D vision sensor is believed to be able to solve this limitation since the object's size and shape can be obtained [21]. The Kinect was chosen in this project since it has 3D depth sensor, relatively fast sampling rate and is available at a low price. The Kinect's 3D depth data is converted into 2D laser scan-like data based on the method proposed in our previous work [21]. Firstly, the 11-bit raw data (which is an array of 640 × 480 elements) was retrieved from the Kinect and converted into real depth. Then, the X and Y coordinates that correspond to each depth pixel (*i.e.*, Z coordinate) were calculated using:

(7) $$X_{i,j}=\left(j-\frac{w}{2}\right)\times \frac{320}{w}\times M\times Z_{i,j}$$

(8) $$Y_{i,j}=\left(i-\frac{h}{2}\right)\times \frac{240}{h}\times M\times Z_{i,j}$$

where *i* and *j* are the pixel's row and column number in the Z-array respectively, w and h are the width and the height of the Z-array and M is the NUI Camera Depth Image to Skeleton Multiplier Constant.

In order to remove the floor that is visible in the 3D data, the pixels located below the height of Kinect were replaced with an infinity value indicating that the data is invalid. The process is repeated for the pixels above the height of the robot since they are not considered as obstacles; thus, the resulting X, Y and Z array only contains the 3D obstacles with which the robot can collide. To convert the data into 2D obstacles (*i.e.*, 2D scan), the minimum element in each column of the Z-array was selected to represent the entire column such that:

(9) $${Z^{\prime }}_{j}=\text{min}(Z_{0,j},Z_{1,j}\ldots Z_{479,j})$$

where j is the respective column number. In other word, the Z′ array (of 640 × 1 elements) contains the Z-coordinates of the nearest obstacle at each horizontal scan angle. Using geometry, the Z-coordinates and the corresponding X-coordinates can be converted into polar coordinate as typically obtained using laser scanner. In contrast to the proposed method, we reduce the Kinect's depth data resolution to QVGA (320 × 240) to minimize processing power and time. All the computations related to image processing (including the conversion into laser scan-like data) were carried out on the netbook onboard the robot. This setup enabled us to minimize the amount of data transferred to the base station; thus retaining a high refresh rate of 60 Hz.

### 3.3. 2D SLAM Techniques

In this paper, two SLAM techniques were used: Gmapping [9,24] and Hector SLAM [7]. Both implementations are available as open source packages in ROS [25,26]. The following subsections describe the underlying principle behind each technique.

#### 3.3.1. Gmapping

The Gmapping algorithm was developed based on the Rao-Blackwellized Particle Filter (RBPF) introduced by Murphy and Doucet *et al.* [27,28]. The RBPF was proposed to solve grid-based SLAM problems and it requires odometry information and the sensor's observations (*i.e.*, scans). The main idea of the RBPF is to estimate the trajectory of the robot, *x*_1:_*_t_* = *x*_1_, …, *x_t_* and the map, *m* given the observations, *z*_1:_*_t_* = *z*_1_, …, *z_t_* and the odometry data, *u*_1:_*_t_*_−1_ = *u*_1_, …, *u_t_*_−1_. This joint posterior is denoted as *p*(*x*_1:_*_t_ m*∣ *z*_1:_*_t_*, *u*_1:_*_t_*_−1_) and can be factorized into Equation (10) through Rao-Blackwellization technique:

(10) $$p\left(x_{1:t},m|z_{1:t},u_{1:t-1}\right)=p\left(m|x_{1:t},z_{1:t}\right).p(x_{1:t}|z_{1:t},u_{1:t-1})$$

The factorization simplifies the computations such that it allows the process to be carried out in two steps. First, the trajectory of the robot can be estimated using the odometry data and the observations. Then, the map (*i.e.*, *p*(*m*∣ *x*_1:_*_t_*, *z*_1:_*_t_*)) can be computed since *x*_1:_*_t_* and *z*_1:_*_t_* are known.

The RBPF uses a particle filter to estimate the posterior *p*(*x*_1:_*_t_*∣ *z*_1:_*_t_*, *u*_1:_*_t_*_−1_), where each particle represents a potential trajectory. An individual map is also computed for each of the particles, since the map is highly dependent on the robot's trajectory. Finally, the particle with the highest probability will be chosen and the associated map will be output by the algorithm.

The Gmapping technique was developed by Grisetti *et al.* [9,24] to improve the performance of the RBPF-based SLAM. The technique consists of an approach to compute an accurate proposal distribution by considering not only the movement of the robot, but also the most recent observation. In other words, the approximation of the posterior *p*(*x*_1:_*_t_*∣ *z*_1:_*_t_*, *u*_1:_*_t_*_−1_) in RBPF was modified to $p(x_{1:t}|m_{t-1}^{\left(i\right)},x_{t-1}^{\left(i\right)},z_{1:t},u_{1:t-1})$, taking into account the previous odometry data and the newest observation. The scan-matcher “vasco” [29,30] is used as to match up the observation against the map constructed so far, thus, providing information on the most likely pose.

The Gmapping method also features an adaptive technique that selectively carries out resampling operations for the particle. A careful resampling step is crucial to avoid the filter from removing good samples (*i.e.*, particles that contain trajectory and map data). The approach proposed by Doucet *et al.*, in [31] was used in Gmapping since it is able to determine the necessity of a resampling step. The approach takes into account the measure of the dispersion of the importance weights, *N_eff_* which indicates how well the particle set approximate the posterior trajectory. The formulation can be denoted as:

(11) $$N_{\text{eff}}=\frac{1}{\sum_{i=1}^{N}{\left({\tilde{\omega }}^{\left(i\right)}\right)}^{2}}$$

where *ω̃*^(^*^i^*^)^ is the normalized weight of particle *i*. The approach suggest that a resampling step should be carried out every time the *N_eff_* drops below half the number of particles, *N* /2. Overall, the implementation was claimed to allow an accurate map learning while reducing the risk of particle depletion.

#### 3.3.2. Hector SLAM

Hector SLAM is an open source implementation of the 2D SLAM technique proposed in [7]. The technique is based on using a laser scan to build a grid map of the surroundings. In comparison to the majority of grid-map SLAM techniques [8,9,24,32], Hector SLAM does not require wheel odometry information. Thus, the 2D robot pose is estimated based on the scan matching process alone. The high update rate and accuracy of the modern LIDAR has been leveraged for the scan matching process thus allowing fast and accurate pose estimates.

The scan matching algorithm used in Hector SLAM is based on the Gauss-Newton approach proposed in [33]. The algorithm seeks to find the optimum alignment of laser scan's endpoints with the constructed map by finding the rigid transformation ξ = (*p_x_, p_y_, ψ*)*^T^* that minimizes:

(12) $$\xi^{*}=\arg\underset{\xi }{\min}\sum_{i=1}^{n}{\left[1-M\left({\text{S}}_{i}\left(\xi \right)\right)\right]}^{2}$$

where the function *M*(S*_i_*(*ξ*)) returns the map value at S*_i_*(ξ) which is the world coordinates of the scan endpoint. Given a starting estimate of ξ, the step transformation Δ *ξ*can be estimated by optimizing the error measure such that:

(13) $$\sum_{i=1}^{n}{\left[1-M\left({\text{S}}_{i}\left(\xi +\Delta \xi \right)\right)\right]}^{2}\to 0$$

Applying first order Taylor expansion to *M*(*S_i_*(*ξ*+ Δ *ξ*) and setting the partial derivative with respect to Δ*ξ* to zero yields the Gauss-Newton equation for the minimization problem:

(14) $$\Delta \xi ={\text{H}}^{-1}\sum_{i=1}^{n}{\left[\nabla M({\text{S}}_{i}\left(\xi \right))\frac{\partial {\text{S}}_{i}\left(\xi \right)}{\partial \xi }\right]}^{T}[1-M\left({\text{S}}_{i}\left(\xi \right)\right)]$$

where:

(15) $$\text{H}={\left[\nabla M({\text{S}}_{i}\left(\xi \right))\frac{\partial {\text{S}}_{i}\left(\xi \right)}{\partial \xi }\right]}^{T}\left[\nabla M({\text{S}}_{i}\left(\xi \right))\frac{\partial {\text{S}}_{i}\left(\xi \right)}{\partial \xi }\right]$$

Although the Hector SLAM does not provide an explicit loop closure approach, the researchers claimed that the system managed to accurately close the loops in many real world scenarios, requires low computation and avoids major changes to the map during runtime. They have also successfully employed the algorithm in Unmanned Ground Robots, Unmanned Surface Vehicles, and Handheld Mapping Devices and logged data from quad rotor UAVs.

## 4. System Overview

**Figure 2.**
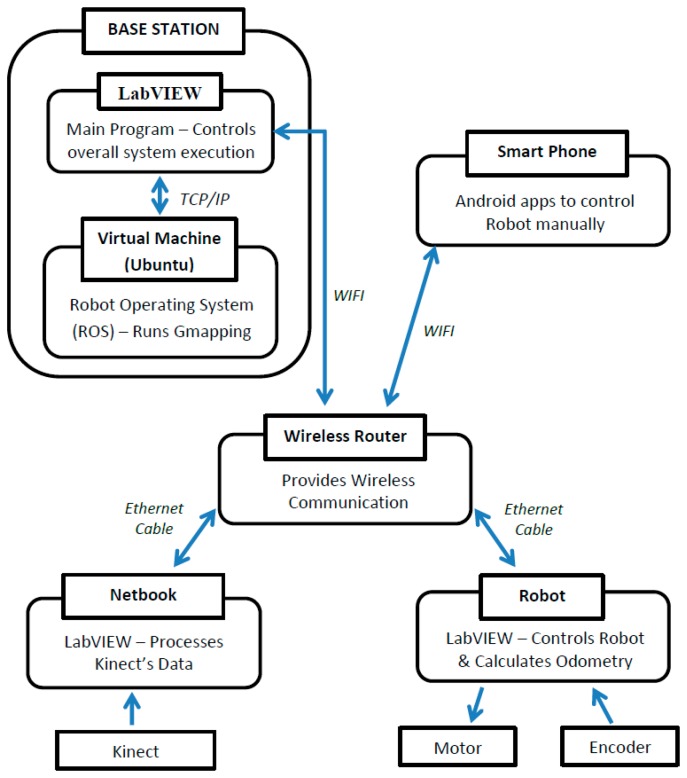
Architecture of the integrated system.

Figure 2 illustrates the overall architecture of the system. The main components of the system are Robot, Netbook and Base Station. A wireless router is used as a medium of data transfer between these three components. The Robot and Netbook are connected to the router using Ethernet cable while the Base Station is connected through WiFi to allow remote monitoring and control. A smart phone equipped with an Android app is utilized for controlling the robot's movement manually and wirelessly.

### 4.1. The Robot

**Figure 3.**
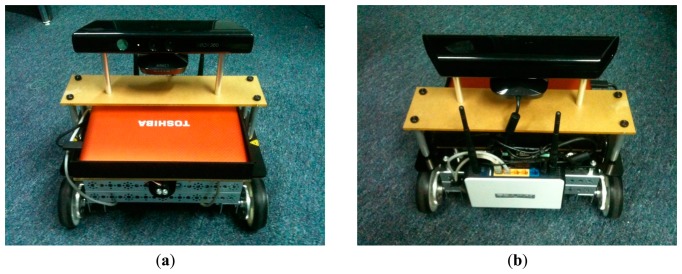
(**a**) Front and (**b**) rear view of the integrated robot.

Figure 3 shows front and rear view of the integrated robot. A National Instruments robot (*i.e.*, NI Robotics Starter Kit 1) was used as the platform of the system. It consists of a single board controller (*i.e.*, sbRIO-9631) featuring a real-time processor, a user-reconfigurable field-programmable gate array (FPGA) and I/O Terminals. The controller controls the robot's motor and processes data from the encoders and other sensors.

### 4.2. The Netbook

The netbook is used to interface the Kinect and perform image processing (see Section 3.2). It consists of a 1.8 GHz Intel Atom Processor and 1 GB Random Access Memory (RAM). The netbook was equipped with the Windows 7 operating system, LabVIEW software and the libfreenect library for interfacing the Kinect.

### 4.3. The Base Station

The base station (*i.e.*, desktop computer) utilizes the Windows 7 operating system with Ubuntu 12.04 installed in a Virtual Machine. The system was designed as such in order to allow simultaneous operation of LabVIEW in Windows and the Robot Operating System (ROS) in a Linux Virtual Machine. The LabVIEW program provides a graphical user interface, controls the whole program execution and provides online monitoring of sensors' data and computed maps. On the other hand, the ROS enables the computation of open-source SLAM algorithms and other useful tools such as ROSbag for recording data. The host (*i.e.*, Windows 7) communicates with the ROS through ROSBridge and the TCP/IP protocol.

### 4.4. The Microsoft Kinect

The Kinect is mounted on top of the robot to provide a wider field of view in the vertical direction. The center of the depth sensor was leveled at 34 cm above the floor. Two pieces of rod were used to fix the Kinect on the robot. The mounting was also carefully designed to align the Kinect in parallel to the robot's body and floor.

### 4.5. The Synchronisation

**Figure 4.**
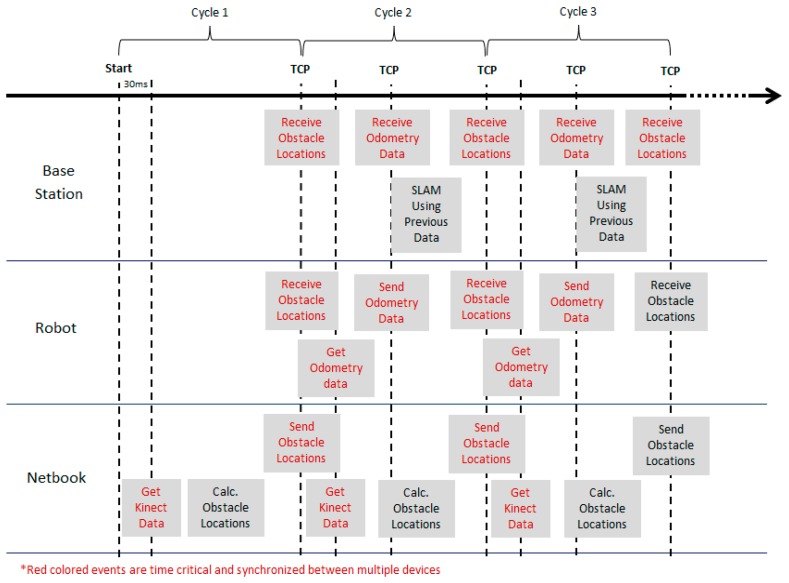
Timeline of synchronization between robot, netbook and base station. The red colored texts indicate the time-critical events which are synchronized between multiple devices.

One significant aspect in developing an integrated system is the synchronisation between multiple controllers. The task becomes more crucial in time-critical applications such as SLAM. False results and unresponsiveness of a system are among the problems that may arise due to synchronisation errors. Figure 4 illustrates the technique employed in this project to achieve synchronization between the robot, netbook and the base station. The TCP/IP function provided by LabVIEW was used as the basis of the implementation.

Initially the Kinect data is acquired by the netbook and processed to obtain a 2D obstacle location (see Section 3.2). Once the defined cycle period is over, the netbook will attempt to transmit this data to the robot and base station which are waiting (*i.e.*, TCP listen) for the transmission request. This information is required by the robot for obstacle avoidance (in auto mode) and also to perform the SLAM calculations in the base station

As soon as the data is transferred, the robot and the netbook will start to acquire new information from the encoder and the Kinect, respectively. Then, the odometry data is transferred from the robot to the base station using the same approach. The remaining period of the cycle is used by the base station to perform SLAM computation using the previous cycle's data and to process any user requests. The processes are repeated again for the next cycle until they are stopped by the user.

## 5. Experimental Environments

**Figure 5.**
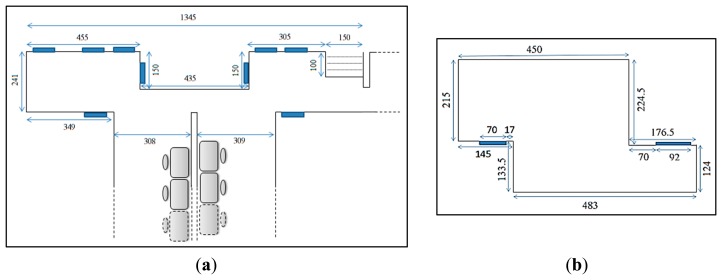
The map of the locations of SLAM experiments in CEASTech building, Unimap. (**a**) is a corridor with features (*i.e.*, desks, chairs and stairs) while (**b**) is the small office room with no features. Dimensions shown are in centimeters.

The experiments discussed in this paper were conducted in two different types of environment in the CEASTech building of the University Malaysia Perlis. The maps of these environments are shown in Figure 5.

(a) Laboratory Corridor (Figure 5a): the corridor has a length of approximately 13.5 m and consists of features such as desks, chairs and stairs. The floor was flat and coated with blue colored epoxy. No high reflection surfaces were observed during the experiment, particularly in the robot's field of view. The lowest step of the stairs has a height of 18 cm which was below the mounting position of the Kinect.
(b) Featureless room (Figure 5b): the room has no features and has a z-shape. The floor was covered with carpet and no reflective surfaces were observed during the experiment.

## 6. Real-Time SLAM

A set of two experiments have been done on each of the environments (*i.e.*, laboratory corridor and featureless room). In each of the experiments, the robot was placed at a different position and orientation. The robot was manually controlled to move randomly without a specific style such as zig-zag or wall following. This setup was preferred as to replicate an automatic SLAM operation in an unknown structured environment where a robot is typically not able to follow a specific style of movement due to the presence of obstacles and a non-uniform building structure.

**Figure 6.**
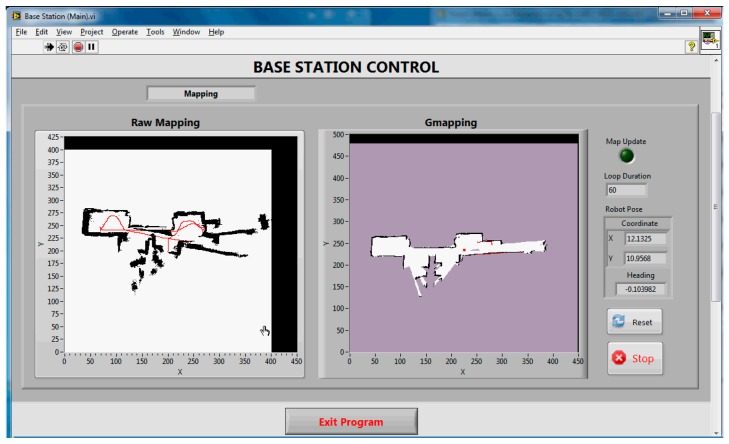
Front panel showing the map of the corridor in Test 1. The red line in the raw map shows the trajectory of the robot based on the odometry data, while the red dot and line in Gmapping map indicate robot pose and obstacle location from the Kinect.

**Figure 7.**
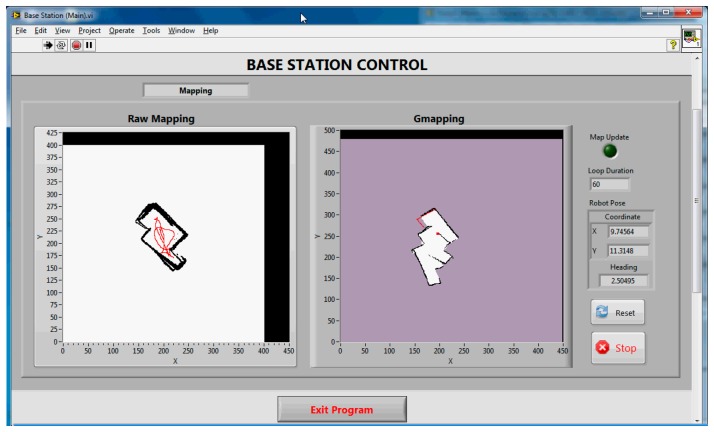
Front panel showing the map of the room in Test 2.

Figures 6 and 7 show the final maps obtained during the real-time SLAM operation for Corridor (Test 1) and Room (Test 2), respectively. Only the Gmapping algorithm was run for the real-time SLAM experiment. The parameters used for the algorithm were modified from the default values determined based on trial and error in previous tests. All the data during the four set of experiments were recorded using the ROSbag tool. This is to allow offline SLAM analysis on both the Gmapping and Hector SLAM. Table 2 shows the duration of each experiment.

Referring to Figures 6 and 7, the left hand side map (*i.e.*, raw map) is the map obtained without any SLAM algorithm; where the odometry and the laser-like Kinect's scan were plotted straight away into the map. The right hand side map is the map obtained from the Gmapping algorithm. The white and black pixels represent unoccupied and occupied areas, respectively, whereas the purple pixels are the unexplored locations.

It can be seen that the Gmapping successfully mapped the corridor with reasonable accuracy. Even though the raw map indicates some misalignments due to odometry errors, the Gmapping managed to correct it. The lowest step of the stairs which was below the Kinect mounting level was also detected and mapped during the test run. The area where the desks and chairs were located was seen to have non-uniform shape since the Kinect only mapped the bottom part of the features. On the other hand, the map obtained using Gmapping for the room (see Figure 7) shows serious misalignment, even though the raw map obtained was quite accurate. This behavior was due to the fact that the Kinect has a narrow horizontal FOV and the room was featureless. The SLAM algorithm failed to scan match correctly and as a result mistakenly determined the pose of the robot during runtime. Detailed explanations and analysis can be found in the Offline SLAM and Analysis Section. The results also proved that the designed system was able to achieve real-time SLAM operation even though it consists of multiple controllers, utilizes wireless connection and more importantly, made use of a virtual machine for SLAM computation.

## 7. Offline SLAM and Analysis

In this section, we analyze the performance of the two SLAM techniques (*i.e.*, Gmapping and Hector SLAM) using the data recorded in the .bag file from the real-time experiments. The ROSbag Play tool was used in order to run the recorded data, simulating the real-time experiment. The parameters of Gmapping and the Hector SLAM were modified in order to see any changes in the performance of SLAM with the Kinect as the only vision sensor. From this point onwards, the four experiments (*i.e.*, Corridor (Test 1), Corridor (Test 2), Room (Test 1) and Room (Test 2)) will be referred to as *Corridor_T1*, *Corridor_T2*, *Room_T1* and *Room_T2*, respectively.

### 7.1. Default Parameters

**Figure 8.**
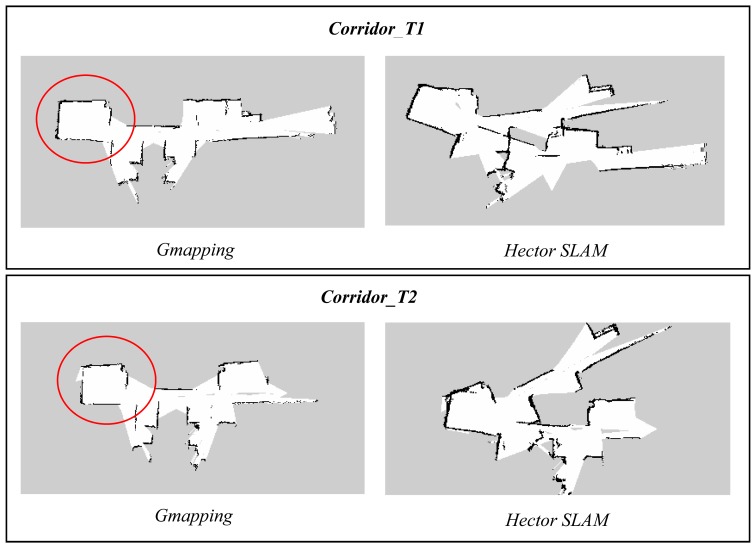
Comparison of maps obtained using Gmapping and Hector SLAM using default parameters.

**Figure 9.**
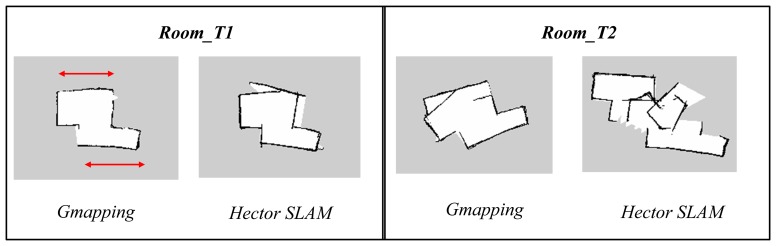
Comparison of Gmapping and Hector SLAM maps obtained using default parameters.

In the first part of the experiments, both the Gmapping and Hector SLAM were executed with default parameters [25,26]. However, since both techniques were developed based on the laser scanner, the parameters specifying the sensor range need to be modified. The parameters were set to 0.6 m to 6 m reflecting the useful range of the Kinect. In addition, the map resolution for both algorithms was made consistent at 0.05 m. Figures 8 and 9 show the results of the experiments.

The maps obtained using the Hector SLAM were inaccurate because the algorithm failed to compute accurate pose estimates. The limited horizontal field of view and range of Kinect affected the performance of the Hector SLAM which only relies on the scans (*i.e.*, scan matching) to estimate the robot pose. The problem gets worse if the area is featureless or has limited features. In addition, it can also be seen that the *Room_T2* map diverged significantly from the real map. This was due to the fact that the Hector SLAM has no loop closing ability and the mapping errors due to the incorrect pose estimates were accumulated over time. This effect is less evident in *Room_T1* since the duration of experiment (see Table 2) was half the duration of the one for *Room_T2*.

The maps obtained using Gmapping are generally more accurate than the Hector SLAM ones when using the default parameters. This is because the Gmapping takes into account the odometry data and that information helps in determining a better pose estimate. However, the same scan matching issue also affected the algorithm's performance. This can be seen in some parts of the corridor and room as indicated by red circle and red arrow, respectively. The areas were mapped as smaller and shorter as compared to the actual dimensions. This occurrence was due to the fact that the areas did not contain any features but only long walls. The shape of the obstacles acquired at each corresponding consecutive scan looks almost the same, causing incorrect scan matching and pose estimation.

### 7.2. Variable Parameters

In this section, we analyze the effect of varying some of the parameters that are believed to affect the SLAM's performance. In each of the specific tests, only the corresponding parameters were changed, while keeping the other parameters consistent with the default values. Tests have been done to all the datasets (*i.e.*, *Corridor_T1*, *Corridor_T2*, *Room_T1* and *Room_T2*). However, only the results for *Corridor_T1* and *Room_T1* are shown in this paper since the main concern was to see the trend of how the map changes.

#### 7.2.1. Gmapping: Number of Particles

**Figure 10.**
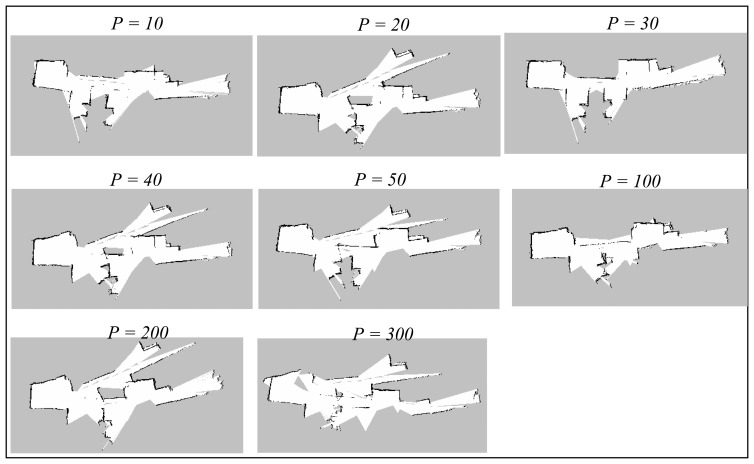
Corridor maps obtained using Gmapping technique with different number of particles.

**Figure 11.**
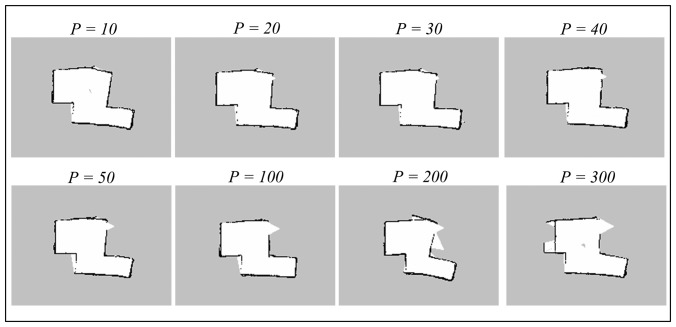
Room maps obtained using Gmapping technique with different number of particles.

The *particles* parameter corresponds to the maximum number of possible trajectories and maps that are kept during the SLAM operation. The trajectory and map with highest probability will be selected and output by the algorithm, so a higher number of particles would generally mean greater possibilities that the output map can converge. The default value of *particles* is 30. The Gmapping algorithm has been run with small number of particles (*i.e.*, 10), up to a large number, 300. Figures 10 and 11 illustrate the results obtained during the experiments.

Referring to both the corridor and room maps, the accuracy seems to be better at lower number of particles, particularly when it is close to the default value, *p* = 30. The maps obtained at high number of particles (*i.e.*, more than 100) diverge significantly from the real measurement since there are too many possibilities that the map can converge to. Thus, any odometry or scan matching error during the SLAM process could lead to serious map inaccuracy.

We also note that the misalignment seen on the corridor maps is originated from the featureless area marked by red circle shown in Figure 8 (Section 7.1). This phenomenon suggests that the algorithm fails to scan match and estimate pose correctly in featureless areas, even the number of particles is set to a higher number. In addition, the map updating period was also observed to be relatively longer for a higher number of particles because the base station has to update each of the particle maps during the process.

#### 7.2.2. Gmapping: Linear and Angular Update

The *linearUpdate* and *angularUpdate* are the parameters that correspond to the required translation and rotation of robot before a scan is processed. The default values are 1.0 m for linear update and 0.5 rad for angular update. Since the range and field of view of the Kinect are limited, we sought to test the Gmapping SLAM with lower linear and angular update values. This will allow a higher rate of scan matching with respect to the change in linear and angular movement of the robot; and perhaps could lower the risk of incorrect pose estimation.

**Figure 12.**
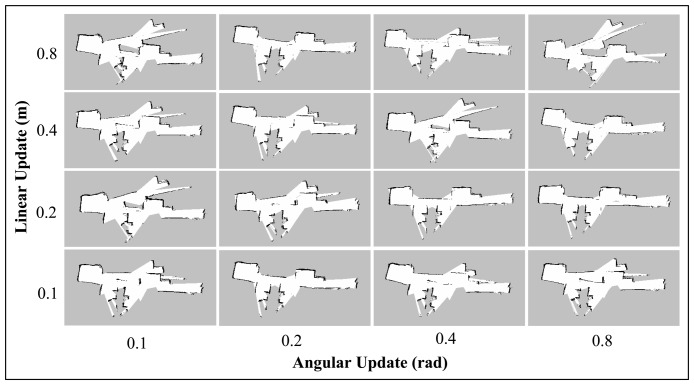
Corridor maps obtained using Gmapping technique with different combinations of Linear and Angular Update values.

**Figure 13.**
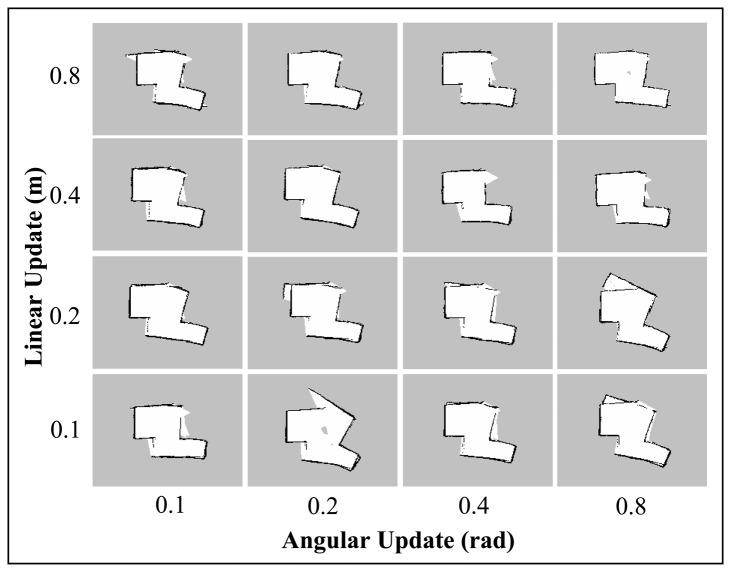
Room maps obtained using Gmapping technique with different combinations of Linear and Angular Update values.

Figures 12 and 13 display the results of varying the linear and angular update values for the *Corridor_T1* and *Room_T1* datasets. As can be seen from the corridor maps, no trend could be inferred for the different linear and angular update values. However, for Room maps, it was found that the higher linear update values improved the convergence to the real map. These results contradict the initial hypothesis of the experiment (*i.e.*, the lower the update values, the more accurate the built map) and suggest that it is difficult to predict the correlation between Gmapping's update parameters and the map quality.

#### 7.2.3. Hector SLAM: Map Update Thresholds

**Figure 14.**
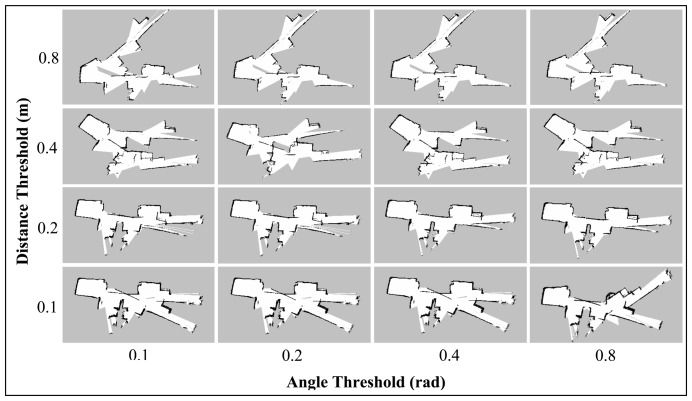
Corridor maps obtained using Hector SLAM technique with different combinations of Distance and Angle Threshold values.

**Figure 15.**
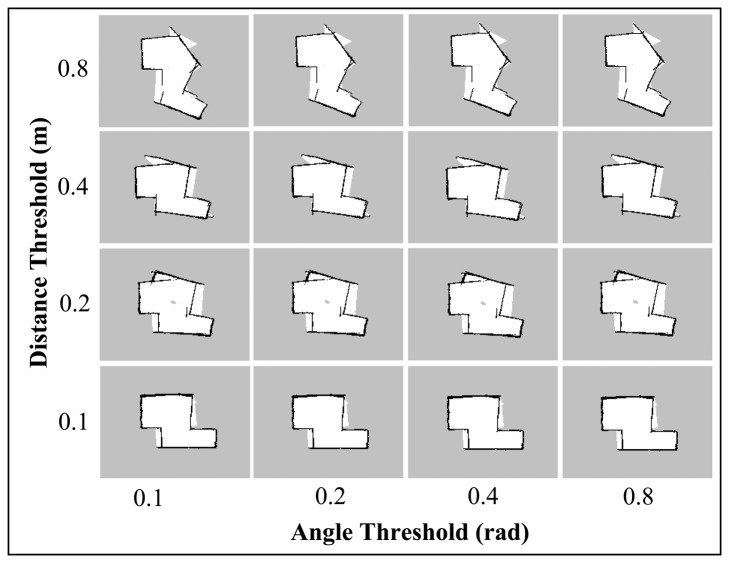
Room maps obtained using Hector SLAM technique with different combinations of Distance and Angle Threshold values.

The *map_update_distance_threshold* and *map_update_angle_thresh* parameters indicate the required distance or angle that the platform has to travel before a map update process is executed. The default distance and angle are 0.4 m and 0.9 rad, respectively. Like the Linear and Angular Update tests for Gmapping (Section 7.2.2), the Hector SLAM was also run with lower update threshold value. This setting may allow the algorithm to provide a map update at a faster rate and thus able to perform better scan matching and pose estimation. Figures 14 and 15 show the final maps when varying these parameters simultaneously.

In both Corridor and Room maps, the effect of the angle threshold parameter was seen to be minimal. Only slight differences could be observed when the angular parameter is changed while keeping the distance threshold constant. On the other hand, the maps for both corridor and room are seen to be more accurate at lower distance threshold values (*i.e.*, 0.1 m and 0.2 m). We also note that the featureless area of the corridor (as indicated in the Default Parameters Section) was mapped accurately using these settings and the maps quality are seen to outperform the ones obtained using the Gmapping algorithm.

## 8. Discussion and Conclusions

It was found that the integrated system has been able to achieve real-time SLAM operation even though the Gmapping was run in parallel on a virtual machine. This proves that it is possible to integrate open-source algorithms (in ROS) into another operating system (such as Windows) for real-time applications. In addition, the implementation provides an easy and reliable way to compare the performance of any Windows-based algorithms with the open-sourced algorithms that are typically implemented in Linux.

The results of the offline experiments suggest that the Gmapping, although having odometry as extra information, often failed to predict correct pose estimates, especially in featureless areas. This phenomenon was due to the fact the Kinect has a relatively limited field of view (as compared to a laser scanner) and is thus unable to provide enough information for reliable scan matching. In addition, it is hard to predict the final map since the Gmapping is based on a particle filter (*i.e.*, probability method) and slight errors in pose estimation could affect the SLAM processes and results significantly.

The modification of certain Gmapping parameters was found to vary the map accuracy. The setting of a lower particle number has been observed to improve the results, especially when the value is close to the default number (*i.e.*, 30). This occurrence is due to the fact that there are so many possibilities that the map can converge to when using high number (*i.e.*, more than 100) and any error in determining the best particle could finally yield a seriously inaccurate map. On the other hand, a lower number of particles is not desirable since it increases the risk of eliminating good particles (*i.e.*, good maps and trajectories). Furthermore, it was found that a higher *linearUpdate* value produces more accurate results in featureless areas, whereas, no trend could be inferred as the *angularUpdate* parameter was varied.

Nevertheless, the determination of optimum set of parameters for the Kinect with the Gmapping technique is a nontrivial process since there are so many parameters available (*i.e.*, more than 30) [25] and it is difficult to predict the correlation between them. Also, the same set of parameters that works in a certain environment, may not work in another type of environment.

The Hector SLAM has been shown to produce similar results where the maps lack accuracy, especially in featureless areas. Again, this is believed to be caused by the limited field of view and range of the Kinect sensor. However, the modifications to the map update threshold parameters to lower values has been seen to significantly improve the map convergence. Interestingly, the corridor maps produced using a *map_update_distance_thereshold* of 0.2 m are relatively accurate, even though the odometry information was not used and the technique only relies on scan registration to estimate the robot pose. Also, the update rate of the map was seen to be faster than with Gmapping since the Hector SLAM is not based on a particle filter and only maintains a single global map. As a consequence, the technique has no loop closing ability.

In conclusion, the Kinect is not a direct replacement for the laser scanner in 2D SLAM applications, mainly due to its relatively limited field of view and range (refer to Section 2 for a specification comparison). Further enhancements include modifications of the SLAM algorithms, application of an adaptive movement strategy or using multiple Kinect sensors to increase the field of view. Another suggestion is to leverage the 3D data from the Kinect's depth sensor to perform scan matching before mapping the obstacles in a 2D map. This implementation may improve the scan matching accuracy and thus provide reliable pose estimates. Nevertheless, the 3D depth data of Kinect has advantages over the 2D laser scanner in terms of detecting and avoiding objects of variable shapes and size. Thus, given that the scan matching and pose estimation issues are solved, the performance of Kinect-based 2D SLAM could be more reliable in a real-world scenario where objects of non-uniform shape exist.

## Figures and Tables

**Table 1.** Technical specifications of Kinect's depth camera as compared to a laser scanner which is typically used in robotics applications, particularly SLAM.

**Components**	**Kinect**	**2D Laser Scanner**
Operating Range (m)	0.4–0.8 to 3.5–6.0	∼0 to 4–250
Horizontal angle (°)	57	180–360
Vertical angle (°)	43	-
No. of Measurement Points	640 × 480 = 307,200	Up to 6000
Approx. costs (USD)	150	1000–15,000

**Table 2.** Duration of each experiment.

**Experiment**		**Duration (s)**
Corridor	Test 1	281
	Test 2	247

Room	Test 1	126
	Test 2	251
